# Effects of wintering under methylmercury exposure on spring reproductive onset in song sparrows (*Melospiza melodia*)

**DOI:** 10.1111/jne.70027

**Published:** 2025-04-21

**Authors:** Claire L. J. Bottini, Calista J. Henry, Scott A. MacDougall‐Shackleton

**Affiliations:** ^1^ Department of Biology The University of Western Ontario London Ontario Canada; ^2^ Advanced Facility for Avian Research University of Western Ontario London Ontario Canada; ^3^ Department of Psychology The University of Western Ontario London Ontario Canada

**Keywords:** avian, hypothalamic–pituitary–gonadal axis, MeHg contaminant, physiology, seasonal transitioning

## Abstract

Exposure to methylmercury (MeHg) on breeding grounds may have numerous deleterious effects on birds, including neurotoxicity, disruption of hormones, and impaired reproduction. But it is unknown if MeHg exposure on wintering grounds can carry over and produce negative effects on the following spring breeding seasonal transition. To evaluate this, we exposed male captive song sparrows (*Melospiza melodia*) to environmentally relevant levels of dietary MeHg for 3 months during winter. We then photostimulated the birds with a long‐day photoperiod and observed them for 21 days post‐exposure. Contrary to our predictions, we found no carry‐over effects of MeHg on the timing of changes in spring reproductive physiology assessed by testes mass, syrinx mass, plasma androgen concentrations, number of GnRH neurosecretory cells, and body condition. However, following photostimulation, MeHg‐exposed birds had smaller cloacal protuberances. Although we observed no obvious effects on the timing of reproductive onset, the results suggest that winter MeHg exposure could induce carry‐over effects on secondary sexual traits that may affect birds' breeding performance. Overall, our findings indicate that songbirds can buffer against the main effects of prior winter MeHg exposure so as to not delay reproductive onset in spring, but more studies are required for long‐term effects on breeding performance.

## Graphical abstract

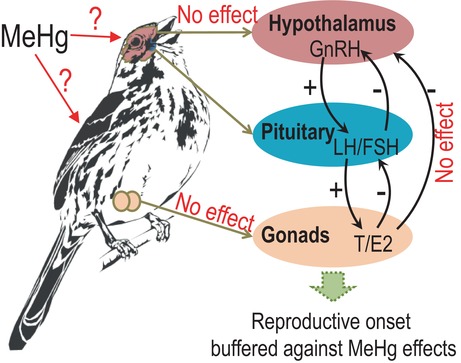

## 1 INTRODUCTION

Organisms alter their physiology to adjust to predictable seasonal or environmental changes. In spring, the increase of day length stimulates numerous neuroendocrine events for birds to transition from winter phenotype to spring reproductive condition. Indeed, this photoperiodic cue directly stimulates the bird's deep brain photoreceptors and intensifies the hypothalamic–pituitary–thyroid (HPT) secretion that in turn promotes the release of gonadotropin hormone and activation of the hypothalamic–pituitary–gonadal (HPG) axis (reviewed in Refs. [1, 2]). This HPG axis stimulation leads songbirds to almost completely regrow their reproductive tract and gonads.^3^, ^4^ Gonadal growth increases the secretion of reproductive androgen and estrogen hormones compared with fall or winter.

Transitions between seasons or annual cycle stages are under multiple time‐sensitive and energetic trade‐offs associated with the modification of physiological functions. For example, a fast transition from wintering into breeding phenotype would allow earlier migratory departure and arrival on breeding grounds, where early arrival is positively correlated with the acquisition of high‐quality breeding territories and reproductive success.^5^, ^6^, ^7^ On the other hand, arriving too soon in a habitat that cannot yet support the bird or its brood could result in reduced survival or breeding performance.^8^, ^9^, ^10^ Moreover, the birds' physiological or energetic state strongly influences the onset and duration of annual cycle stages. For example, female greater snow geese (*Chen caerulescens atlantica*) with high pre‐migratory condition had an earlier egg laying date than those in low condition.^11^ Also, high testosterone levels hasten the transition from winter to breeding but negatively affect fat stores.^12^ Therefore, any event or compound that perturbs either the timing or homeostasis of birds could affect their transition between annual cycle stages and ultimately influence their performance.

Methylmercury (MeHg) is a ubiquitous neurotoxin^13^, ^14^, ^15^ whose levels are predicted to increase with global warming.^16^, ^17^, ^18^ MeHg comes from the conversion of inorganic mercury (coming from both natural and anthropogenic sources; reviewed in Refs. [19, 20, 21]) in its toxic methylated form by bacterial activity in anaerobic conditions.^22^, ^23^, ^24^ Once produced, MeHg enters the food chain via aquatic and soil invertebrates (reviewed in Refs. [25, 26, 27]) until reaching high concentrations in top predators such as birds.^28^, ^29^, ^30^ Exposure to MeHg can negatively affect a bird's energy balance,^31^, ^32^, ^33^ hormone activity (reviewed in Ref. [34]) and reduce its reproductive performance (reviewed in Ref. [35]). Birds exposed to MeHg on the breeding grounds have reduced clutch or brood size.^36^, ^37^, ^38^ Such outcomes are often assumed to be caused by embryo toxicity,^39^ though MeHg can also affect parents, with changes in reproductive behavior^40^, ^41^, ^42^ or in energy stores to invest in reproduction.^43^, ^44^, ^45^ Additionally, MeHg exposure can carry over to affect birds at later times. For example, MeHg winter exposure can carry over and affect summer total mercury (THg) concentration in adults,^46^ and developmentally exposed birds will have reduced reproductive output once adults.^47^ Hence, it is often not clear if the effects on breeding physiology and behavior reviewed above are directly caused by exposure at the breeding site or if prior exposure could affect reproduction via carry over effects.^48^, ^49^

In this study, we aimed to assess if MeHg exposure on wintering grounds could affect songbirds' seasonal transition to their breeding phenotype. We used a migratory subspecies of song sparrows (*Melospiza melodia melodia*) that we exposed during three winter months to environmentally relevant dietary levels of MeHg that are known to induce reproductive deficits in other studies. While during the nonbreeding period song sparrow mostly eat seeds, fruits, and some invertebrates depending on availability along water courses,^50^ this species is a good model for other migratory songbirds consuming insects during winter. Following exposure, the MeHg treatment was stopped and birds were photostimulated and euthanized 24–26 days later to assess their progress in transitioning toward reproduction. We hypothesized that MeHg could affect breeding onset through physiological disruptions and increased energetic costs that may carry‐over after the exposure period. We predicted that onset of reproductive condition (GnRH neurosecretory cells, testosterone levels, gonad size, and cloacal protuberance) would be delayed in MeHg‐exposed birds. We also predicted a potential time by treatment interaction, under the hypothesis that the birds can recover from MeHg harm after a time without exposure, and/or the physiological changes associated with photostimulation promote the appearance of latent MeHg effects not detected previously. Furthermore, we quantified birds' body condition to document the potential MeHg effects on energy stores during the experiment. For practical reasons, the birds' singing behavior was not quantified, while the brain song nuclei volume data and other hormonal measures will be reported in a future study, as those data are beyond the scope of the present manuscript.

## 2 METHODS

### 2.1 Bird capture and housing

Between 2 September and 24 September 2019, we used mist‐netting and song playback to capture 33 song sparrows (5 females, 26 males, 2 unknown) near Port Rowan, Ontario (42°37′19.2″N 80°27′44.4″W), the University of Western Ontario campus (43°00′24.5″N 81°17′14.0″W), and the university observatory grounds near Elginfield, Ontario (43°11′40.0″N 81°18′34.2″W). All birds were captured under permission from the Canadian Wildlife Service, Environment and Climate Change Canada (Scientific Permit CA0244). Soon after capture, we transported the birds to the Advanced Facility for Avian Research (AFAR), located at the University of Western Ontario. Birds were housed indoors, at 20°C–22°C, in individual cages. All animal procedures were approved by the University of Western Ontario Animal Care Committee (protocol no. 2017‐161). A few days after capture, three birds unexpectedly died or were euthanized, while a fourth bird (control treatment) died from unknown causes during the course of the experiment in early January.

For the first several weeks following capture, each of the remaining 29 birds were kept under the same conditions of natural autumn declining photoperiod (light schedule was updated every week). On the 3 and 18 October, all birds received a sub‐cutaneous injection of anti‐parasitic treatment (ivermectin, 0.025 μL injected as concentration of 0.2 mg/mL). On 9 December 2019 we changed the light schedule to a constant winter photoperiod of 9 h light and 15 h dark (9L:15D). This light schedule remained consistent throughout the entire treatment exposure and was then switched to a summer photoperiod condition (16L:8D) at the end of the treatment exposure period on 2 March 2020. This summer photoperiod remained consistent throughout the post‐exposure period of the experiment. We chose to use immediate photostimulation rather than gradual increase in day‐length to give all of the birds a synchronized cue for the development of reproductive functions, as commonly done in studies on avian photoperiodism.^51^, ^52^

Under all circumstances, birds were kept with ad libitum access to water and food. During the first 8 weeks after their capture, the birds' diet was a mix of seed (Living World Premium Mix for Budgies parakeet seed) and ground Mazuri (small bird diet). This long pre‐experimental period allowed birds to habituate to captivity and to complete their feather molt before the experiment started. Indeed, this species only molts once during autumn, ranging from July to November^50^, ^53^, ^54^ and we also regularly checked that no further molt affected the birds' MeHg exposure during this study. On 18 November, we began gradually changing their diet to a nutritionally complete agar‐based synthetic diet by mixing the seed with the synthetic diet. The agar‐based diet dry mass contained 60% carbohydrate, 13.4% protein, and 10.6% lipid (Table S1; Text S1). This diet became the birds' main food starting on 30 November until the end of the experiment. We replaced the food every day with 25 g of freshly prepared synthetic diet, and once a week, we gave the birds an additional 5 g of uncontaminated blended chicken eggs.

### 2.2 MeHg treatment exposure

**FIGURE 1:**
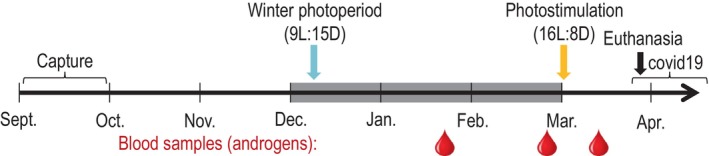
Experimental timeline in 2019–2020. The grey rectangle indicates the period of MeHg exposure (from 2 December 2019 to 1 March 2020). We took an initial blood sample from all birds on 25–28 September to confirm the birds' initial low THg levels. The red drop symbols indicate dates of blood sample collection for testosterone measurements (on 23–24 January, 27–28 February, and 12–13 March 2020, respectively). We also measured body mass every 2 weeks (not represented in the figure). The COVID‐19 bracket indicates how the pandemic and university closure forced us to terminate this study sooner than expected.

Each bird's sex was first identified by polymerase chain reaction (PCR) of sex‐specific genetic markers, and later confirmed by gonad extraction post‐euthanasia. We pseudo‐randomly assigned the birds into one of two experimental groups, balancing for sex: 14 (3 females, 11 males) control birds received uncontaminated agar‐based diet, while 15 (2 females, 13 males) birds were exposed to MeHg through their agar‐based diet. We started the birds' MeHg exposure on 2 December 2019, with the diet treated with methylmercury chloride (MeHgCl) at 0.22 ± 0.017 mg kg^−1^ (mean ± *SD*) wet weight (ww), corresponding to 0.68 mg kg^−1^ dry weight. We chose this dose to be close to environmentally relevant levels measured in insects in contaminated areas (e.g., ranging from 0.019 to 2.72 mg kg dw^55^, ^56^, ^57^), but at the same time to be within the range of exposure high enough to induce reproductive dysregulation. Indeed, the effective concentration threshold resulting in a 20% decrease in reproduction (EC_20_) was 0.16–0.75 mg kg^−1^ ww in the diet of small‐medium bird.^35^ Previously, a dose of 0.19 mg kg^−1^ resulted in several physiological effects on hormones, molt, and migratory behavior in the same species.^49^, ^58^ For each batch of food made, a 1 g sample was collected for later THg analysis. The birds were maintained under control or MeHg diet treatments for 3 months (91 days). On 2 March 2020, corresponding to the first day of photostimulation, all birds received only the uncontaminated agar‐based diet until the end of the study. We monitored the birds for an additional 24–26 days post‐exposure before they were euthanized. The timeline of experimental treatment and manipulation is illustrated in Figure 1.

For safety reasons, the experimenters were not blind to the treatment during procedures requiring handling (i.e., animal care, blood and tissue collection, mass and cloacal measurements, euthanasia) and statistical analysis, but were blind during samples processing (i.e., tissue mass, androgen and GnRH laboratory and microscope analysis).

### 2.3 Blood collection, MeHg and androgen analysis

We took an initial blood sample from all birds on 25–28 September 2019, before the experiment started to confirm the birds' sex and initial low levels of THg. Starting on 9–10 January 2020, we took blood samples every 2 or 4 weeks (Figure 1). All blood sampling occurred in the morning between 9:00 and 11:00 am. To collect blood samples, we punctured the birds' wing vein with a needle and collected 50–200 μL of blood into heparinized microhematocrit tubes. If samples could not be centrifuged quickly, they were kept on ice or in a refrigerator. Within 5–90 min of collection, samples were centrifuged to separate plasma from red blood cells. Samples were then stored at −80°C for several months and moved to −30°C in September 2020 until analysis. We used plasma samples for hormone analysis (androgen) and red blood cells for THg measurement.

Methods for THg analysis of food and blood samples are provided in Ref. [53]. Specifically, for these samples, the mean recovery of certified reference material (DORM‐4—fish protein; National Research Council Canada, and CRM PACS‐3—Marine Sediment Reference Material) was at 102%. The aqueous calibration check standard (CCS) and CCS duplicate were at 101% while the initial and ongoing precision and recovery were at 102%. The relative percent difference between duplicate samples was 3% for both food and blood while the relative difference between spiked duplicates was at 2%. The method detection limit was 0.07 ng and the method reporting limit was 0.22 ng.

Plasma androgen concentrations were assessed via an ELISA kit (Salivary Testosterone ELISA Kit, Salimetrics Assay no. 1‐2402), following the kit protocol. This assay has been validated for this species^59^ and a variety of other songbird species,^60^ but partially cross‐reacts to several other androgens (i.e., Dihydrotestosterone at 36.4%, 19‐Nortestosterone at 21.02%, and Androstenedione at 1.157% according to the manufacturer's information); hence, we used the term “androgen” instead of “testosterone” to be more accurate. Samples were diluted in testosterone assay diluent depending on their collection time: winter samples had 20 μL of plasma mixed with 55 μL of diluent, while post‐photostimulation samples had 15 μL of plasma mixed with 60 μL of diluent. Inter‐assay coefficient of variation was determined from a homogenized pooled sample along with high and low control samples analyzed on each plate. The inter‐assay and intra‐assay coefficient of variation (CV) were 4.2% and 5.1% (*n* = 93), respectively. Detected concentrations of all samples were within the standard curve except for one individual with a very high concentration. This sample was then attributed the concentration of the highest standard.

### 2.4 Body condition

Before the experiment, we measured each bird's tarsus length to the nearest 0.1 mm using dial calipers. Upon arrival and then once every 2 weeks starting on 2 October 2019, we measured each bird's body mass with an electronic balance to the nearest 0.01 g. We used change in total body mass through time as a proxy for change in body condition for statistical analyses. Indeed, in contrast to a prior study,^54^ we did not find a significant effect of tarsus length on male birds' mass (linear mixed effect model with birdID as random intercept: tarsus *p* = 0.165). This may be due either to seasonal effects, to the lower size variation between males (tarsus min–max: 20.7–22.2 mm) or due to the exclusion of females from this study dataset.

### 2.5 Cloacal protuberance

To assess morphological changes associated with breeding, we measured the birds' cloacal protuberance size during their regular health checks, on 18 February (about 2 weeks before photostimulation), 4 March (2 days after photostimulation), and 21–22 March (19–20 days after photostimulation). In male songbirds, the cloacal protuberance is involved in sperm storage and delivery^61^, ^62^, ^63^ and the swelling changes seasonally in response to photoperiod and androgen levels, reaching its largest size soon after arrival at the breeding site,^64^, ^65^, ^66^ thus its volume is indicative of reproductive status. The length and width of the cloacal protuberance were measured using dial calipers to the nearest 0.5 mm. The volume was then calculated using the equation for an ellipsoid:

(1) $$\left(\frac{4}{3}\times \pi \times {\left(\frac{\text{width}}{2}\right)}^{2}\times \frac{\text{length}}{2}\right)\times 1000.$$

### 2.6 Euthanasia and tissue collection

On 25–27 March 2020, a bit more than 3 weeks following photostimulation and post‐exposure, we euthanized the birds using an overdose of isoflurane inhalation, followed by a transcardial perfusion with 0.1 M phosphate buffered saline (PBS; pH = 7.4) followed by 4% paraformaldehyde. We removed the brains and submerged them in paraformaldehyde for 48 h, then transferred them into 30% sucrose in PBS for an additional 48 h for cryoprotection. On the fourth day post‐euthanasia, we quickly weighed the brains with an electronic scale to the nearest 0.1 mg and immediately froze them under crushed dry ice before being stored at −80°C for long‐term storage.

Gonads, syrinx, and liver were also dissected out immediately following brain removal and submerged in 4% paraformaldehyde during 7–10 days before being weighed and discarded. Liver has an important role in energy balance and contaminant depuration and is known to be affected by MeHg exposure.^67^, ^68^, ^69^ A change in liver size may indicate a chronic toxicity that could explain some physiological differences compared with uncontaminated individuals. Syrinx mass increases in response to rising circulating androgens in spring^70^, ^71^ and is thus another indicator of breeding state development.

### 2.7 Brain processing and immunochemistry

We used a cryostat at −20°C to cut brains into 40 μm coronal sections. Sections were collected in alternating series. From the division of the tractus septomesencephalicus (TSM) to the anterior commissure, we collected every second section for GnRH staining.

We followed a 3‐day free‐floating immunohistochemistry protocol for GnRH staining. The brain sections were washed twice in 0.1 M PBS, then placed for 15 min in a 0.5% solution of hydrogen peroxide (no. 216763, Sigma‐Aldrich) diluted in PBS to reduce endogenous peroxidase. Following this, sections were washed an additional three times in 0.1 M PBS before being incubated in 10% normal goat serum (no. S‐1000‐20, Vector Laboratories) mixed in 0.3% phosphate‐buffered saline with triton (PBS/T) overnight at 4°C. On the second day, sections were incubated in a polyclonal primary antibody labeling GNRH1 (ImmunoStar Cat no. 20075, RRID: AB_572248) diluted in 0.3% PBS/T. This antibody has been validated and used in a variety of mammals, fish, and poultry. Trays were then incubated for a 1 h at room temperature with agitation on a shaker before being placed at 4°C to incubate for 24 h.

Finally, the addition of the secondary antibody and staining visualization occurred on the third day of the procedure. We first washed the sections three times in 0.1% PBS/T, before incubating sections in 1:250 biotinylated anti‐rabbit IgG made in goat (no. BA‐1000, Vector Laboratories) secondary antibody for 1 h with agitation on a shaker. We washed the sections another three times in 0.1% PBS/T, and then incubated them for 1 h in avidin‐biotin horseradish peroxidase complex (ABC; Vector Laboratories Elite Kit, no. PK‐4000). After two washes in 0.1% PBS/T, sections were visualized with diaminobenzidine solution (DAB; Vector Laboratories, no. VECTSK4103). Sections were then mounted onto microscope slides (Fisherbrand Superfrost Plus, no. 12‐550‐15), and then serially dehydrated in ethanol, cleared in xylene substitute solvent (SafeClear, no. 23‐314629, FisherBrand), and protected with coverslips using Permount (no. SP15‐500, Fisher).

### 2.8 Microscopy and count of GnRH producing cells

**FIGURE 2:**
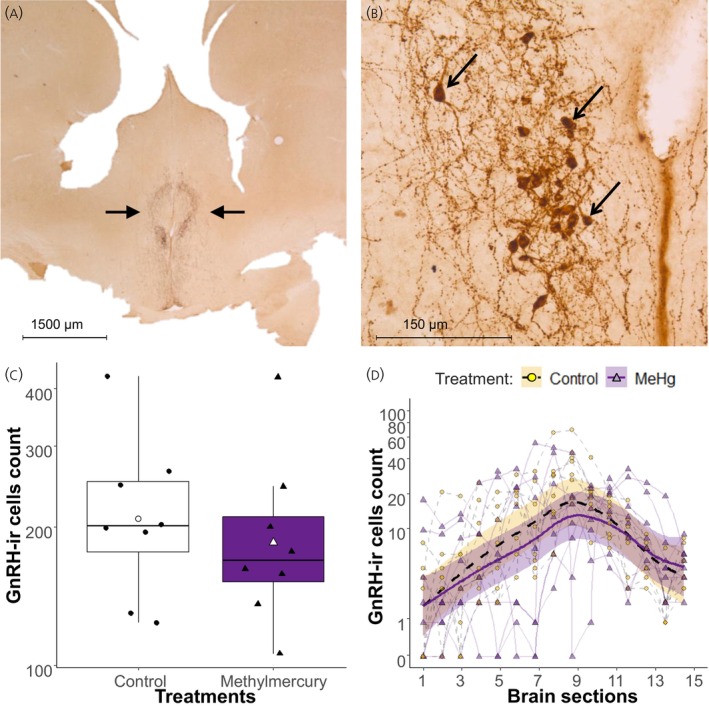
Summary of microscope measurements for GnRH‐ir cells in the preoptic area of the hypothalamus. (A) Example of GnRH‐ir section for general position of the areas of interest (within the black arrows). (B) Example of GnRH‐ir cells (indicated by arrows) that were counted, and immunoreactive fibers. (C) Male birds' GnRH‐ir cells count according to treatment. Boxplots indicate 25th, 50th, and 75th percentiles and whiskers indicate range, with individual horizontally jittered data points overlaid. The boxplot color and symbols' shape indicate the individual's treatment, where the control group is indicated by black circles in white boxplot (*n* = 8), and the MeHg exposed group is indicated by black triangles in purple boxplot (*n* = 8). The white symbols indicate the mean count for each treatment group. (D) Change in GnRH‐ir cells count with brain sections and treatment group. Brain section 1 corresponds to the 12th section rostral to the anterior commissure, section 13 is the first section where the anterior commissure appears and section 15 correspond to the 2nd section caudal to the anterior commissure. Nonparametric splines were fitted with the predictions of the GAMM models at 10 knots and 95% confidence interval. Lines type and colors as well as symbols shape and colors indicate the treatment groups, where the control group is represented by dashed line and empty circles (*n* = 8), and the MeHg exposed group is represented by solid line and purple triangles (*n* = 8). Connected symbols represent individual bird ID.

All photomicrographs and measurements were taken by an observer blind to the treatment conditions. We aimed to calculate the number of GnRH‐immunoreactive (GnRH‐ir) cells in the preoptic area of the hypothalamus. GnRH‐ir slides were examined using a bright‐field light microscopy (Zeiss Axiophot microscope) with a digital camera module (SPOT Idea 5.0 Megapixel camera, Model no. 28.2). Each microscope slide was analyzed using a 5× objective lens. The observer counted the total number of visible GnRH‐ir cell bodies in the preoptic area of the hypothalamus (Figure 2) in each bird. Cells were counted in 15 sections, beginning from the 12th section rostral to the anterior commissure through to the 2nd section caudal to the anterior commissure. Because of technical issues when processing the tissue, some sections were lost or damaged. For each hemisphere where cell counts were not possible (65/840 hemispheres), the number of cells was estimated by averaging the cell counts from the sections' hemisphere on either side or by using the cell count from the opposite hemisphere when it was intact. However, to avoid biasing the results with estimated data, the birds with more than three estimated hemispheres (10% of estimated data) were later removed from the dataset, resulting in a sample size of 8 control and 8 MeHg exposed male birds. Statistical models with the removed birds resulted in qualitatively similar results.

### 2.9 Statistical analysis

All statistical analyzes were conducted in R Version 4.0.3 (http://www.Rproject.org/).^72^ For every result where time was a variable, we fitted two models corresponding to our two hypotheses: (1) main effect of MeHg and/or time without interaction, (2) interactive effects of MeHg and time. All preliminary checks to set up the model parameters were done on the more complex model with the interactions, and in the manuscript we presented the most parsimonious models, while the second models are presented in SI. All results are presented as arithmetic means ± *SD*, and level of significance set at *p* < 0.05. Assumptions of model normality and homogeneity of residuals were checked. Due to the low sample size of females (*n* = 5), we did all analyses on males only; more information on the sex differences is available Ref. [58] in the freely available dataset and R code (Mendeley data, doi: 10.17632/4sdmfy98xc).

#### 2.9.1 Androgen analysis

To test for seasonal and/or treatment effects on plasma testosterone concentration, we first log‐transformed the data and ran a generalized linear mixed model (glmm) via the package lme4 (https://CRAN.R-project.org/packages=lme4),^73^ using a gamma distribution without link specification. Simpler models such as linear mixed effect model (lme) or untransformed data with glmm models and other family specifications led to worse residual distribution and singularity issues (more details provided in accessible R code). We performed preliminary checks to identify the best random structure (using AICc and the *anova* function to compare models) and to assess if variables with no a priori expectation (i.e., mass, tarsus size, or cage position) were influencing the data and needed to be included or not in the model. This step served to identify influential variables for our data while not oversaturating the models we were working with. Preliminary checks indicated no influence of mass (anova comparison of model with or without this variable: *p* = 0.852), tarsus size (*p* = 0.214) or cage position (*p* = 0.728) that were thus not included in our main model.

Following these preliminary analyses, the model fixed effects included the month of plasma collection, treatment, and the interaction between treatment × month. Bird ID was added as a random effect to account for repeated measures. We then determined significant effects with a Tukey post hoc test using the *glht* function. Fixed effects 95% confidence intervals were extracted with the *confint* function of the stats package (https://CRAN.R-project.org/package=STAT),^74^ using wald method specification. Model *R* ^2^ value was extracted via the *rsq.glmm* function of the rsq package (https://CRAN.R-project.org/package=rsq).^75^

#### 2.9.2 Body condition

To analyze the variation of male birds' mass (as an index of body condition) and evaluate its non‐linear relationships with time for each treatment group, we used generalized additive mixed model (GAMM) from the *mgcv* package (https://CRAN.R-project.org/package=mgcv).^76^ For this, we used a Gaussian function of bird's mass evaluated for treatment as a grouping factor and week as a smooth term set with a B‐splines, as well as the factor smooth interaction of week by treatments. To account for autocorrelation between the repeated measurements across time, we added an autocorrelation structure of order 1, with the function *corAR1* on the week covariate. To account for repeated measures and temporal variability in bird mass, we included birdID as a random intercept. The spline and random structures were chosen after preliminary checks showing a better model's AIC value when compared with other possible model structures (e.g., selected model AICc = 1110.67; model with BirdID and week as random correlated intercept and slope AICc = 1136.73; two random intercept model AICc = 1294.31; combined two intercept and a slope model AIC = 1264.64). The model results were obtained by fitting the model with restricted maximum likelihood.

#### 2.9.3 Cloacal protuberance

To test for seasonal and/or treatment effects on cloacal protuberance, we first calculated the percentage change in cloacal protuberance volume from the first measure on 18 February. We then square root transformed the response variable and used a lme model including treatment, factorial week of measure, and their interaction as fixed effects and bird ID as a random effect to account for repeated measures. However, in this model the treatment fixed effect had different results interpretation between the summary function (looking at the difference between MeHg birds versus control birds; *p* = 0.31) and the anova function (looking at the overall effect of treatment variable on the response variable; *p* = 0.044). Such a difference may imply that the presence of an interaction obscured a treatment effect by reducing statistical power. We therefore decided to simplify our model by removing the interaction, thus testing our first hypothesis. The model with the interaction is presented in Table S4.

#### 2.9.4 Tissue mass and volume (testes, syrinx, brain, and liver)

To assess the treatment effects on organ mass, we first log‐transformed the testes and liver mass data. For testis, syrinx, and brain mass, we analyzed the effect of treatment using a *t*‐test after validating the data normality and homogeneity. We also used an lm on the log‐transformed liver mass as the dependent variable with treatment and body mass at euthanasia as independent fixed effects. We chose this model because liver mass may represent up to 5% of the bird's body mass (mean ± *SD*: 0.90 ± 0.20 g in birds of 21.90 ± 1.87 g on day of euthanasia) and both measures are linearly correlated (Spearman correlation test: *S* = 1012, rho = 0.56, *p* = 0.0051) independently of bird's size (Spearman correlation between liver mass and tarsus size: *S* = 2519.1, rho = −0.095, *p* = 0.66). Hence, we use body mass as a covariate in the model to account for other physiological parameters that may influence the effect of treatment on liver mass.

#### 2.9.5 Brain GnRH

To test the effect of treatment on the number of GnRH‐ir cells in male birds, we first log‐transformed data to conform to the test requirements of normality and then performed a *t*‐test.

A prior study demonstrated that photoperiodic changes in reproductive state, castration, and female presence resulted in region‐specific alterations in the number of cells expressing GnRH in male starlings.^77^, ^78^ Hence, in a second step, we assessed if MeHg exposure may affect the distribution of GnRH‐ir cells in specific regions of the hypothalamus, using the coronal section number to assess variation along the rostral‐caudal axis. For each section, the total cell count of each hemisphere was summed to remove this parameter's variability from the model and reduce the need for a hierarchical nested model. Because the cell count is not linearly distributed across brain sections, we chose to use GAMM to better handle the data variation. We used a GAMM on the log + 1 transformation of the GnRH‐ir cells count and a Gaussian function. The model explanatory variables included the treatment as a grouping factor and the brain section number as smooth terms set with a cubic regression spline, as well as the factor smooth interaction of brain section × treatment. The autocorrelation between section numbers was handled with the *corARMA* function, setting the autoregressive order and the moving average order with values of 2 and 0, respectively. These values were selected after AIC comparison of models with values varying between 0 to 5 for each component, and such model structures resulted in better AIC than models with autocorrelation managed with the *corAR1* function. The bird ID was included in the model as a correlated random intercept that, based on model AIC, performed better (AIC = 601.90) than models with other random structures, such as random intercept and slope (AIC = 606.32), two random intercepts (AIC = 655.91; note that in mgcv package, this gives the same results as a nested structure where section is number nested within birdID), and a model combining random slopes and two random intercepts (AIC = 624.88). This random intercept structure accounts for the repeated measures, while the hierarchical structure of section number being nested in bird ID is accounted for by the corARMA function. The model results were obtained by fitting the model with restricted maximum likelihood.

## 3 RESULTS

### 3.1 THg analysis

On 25–28 September, all birds had a whole blood THg of 0.0076 ± 0.014 mg kg^−1^ (mean ± *SD*; *n* = 30). Throughout the experiment, control birds had a THg concentration in red blood cells of 0.051 ± 0.065 mg kg^−1^ ww. On the contrary, MeHg‐exposed birds had a red blood cells level of 10.85 ± 1.24 mg kg^−1^ at the end of exposure on 27–28 February 2020 and of 5.22 ± 1.93 mg kg^−1^ at the end of the experiment on 25–27 March 2020. Because in this species whole blood THg level is equivalent to approximately 48.8% of red blood cell levels (Bottini et al., unpublished data), MeHg‐exposed birds would have a whole blood equivalent THg level of ~5.29 mg kg^−1^ at the end of the exposure period.

### 3.2 Plasma androgen analysis

**FIGURE 3:**
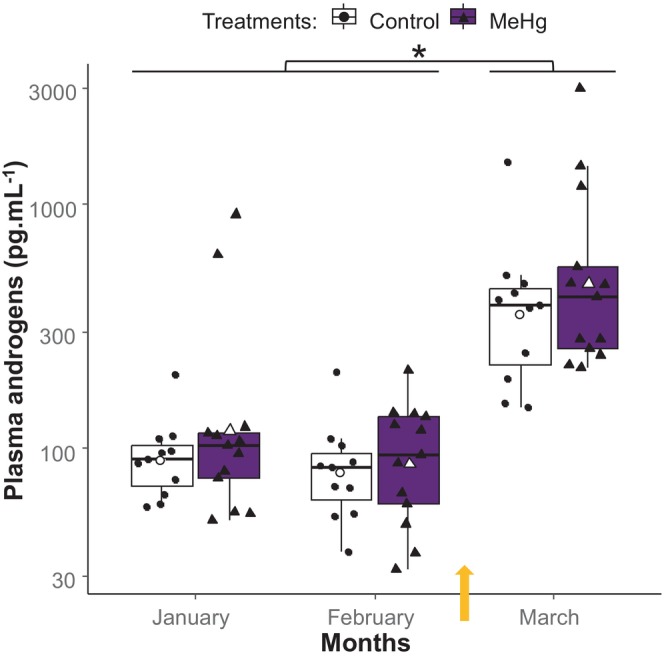
Plasma androgens concentration (pg mL^−1^) on log scale, in relation to month and MeHg exposure. Boxplots indicate 25th, 50th, and 75th percentiles and whiskers indicate range, with individual horizontally jittered data points overlaid. The boxplot color and symbols' shape indicate the individual's treatment, where the control group is indicated by black circles in white boxplots (*n* = 11), and the MeHg exposed group is indicated by black triangles in purple boxplots (*n* = 13). The white symbols indicate the mean testosterone for each treatment group. The yellow arrows indicate the end of exposure and change in photoperiod. The asterisk indicates that male birds show significant increase of testosterone in March (Tukey post hoc test, *p* < 0.01).

Androgen concentration was not affected by treatment or the interaction of month × treatment (Table S2), but was affected by the month of measurement (Table 1). Male samples collected post‐photostimulation on March 12–13 had a higher concentration of androgens than prior time points (Tukey post hoc test, *p* < 0.01; Figure 3). The two time points during exposure were not different from one another (Tukey post hoc test, *p* > 0.1).

**TABLE 1:** Result of glmm model on plasma testosterone concentration, corresponding to our first hypothesis (main effects only).

Plasma testosterone levels variation					
*Predictors*	*Estimates*	*SE*	*95% CI*	*Statistic*	*p*
**(Intercept)**	**0.22**	**0.0069**	**0.21 to 0.23**	**32.13**	**<0.001**
Treatment [MeHg]	−0.0084	0.0073	−0.023 to 0.0060	−1.15	0.25
Month [February]	0.011	0.0071	−0.0030 to 0.025	1.54	0.13
**Month [March]**	**−0.049**	**0.0062**	**−0.061 to −0.037**	**−7.93**	**<0.001**
***Random effects***	***Variance***	***SD***			
Bird.ID *τ*_00_	<0.001	0.0086			
Residuals *σ* ^2^	0.014	0.12			
Intraclass‐correlation coeff. (ICC)	0.27				
Observations/*N*	72/24				
Marginal *R* ^2^/Conditional *R* ^2^	0.54/0.59				

*Note*: Indication of [MeHg] and [month] signals which factor group of the data is compared with the reference group (e.g., control treatment or month of January). Final model's significant variables (*p* < 0.05) are displayed in bold. Results from the model including the treatment × month interaction is presented in Table S2.

### 3.3 Body condition

Body mass variation was not affected by treatment or the interaction of week × treatment (Table S3), but was affected by the main effect of week (Table 2). Birds' mass increased upon arriving in captivity, then stabilized during the treatment exposure period before decreasing post‐photostimulation (Figure S1).

**TABLE 2:** Result of GAMM model on bird's mass variation.

Bird's mass variation				
*Parametric coefficients*	*Estimates*	*SE*	*t value*	*p*
**(Intercept)**	**23.28**	**0.23**	**101.25**	**<0.001**
Treatment	0.10	0.32	0.30	0.77
*Smooth parameters*		***edf***	***F value***	***p***
**week**		**10.16**	**30.54**	**<0.001**
***Random Effects***	***Intercept***	***Residual***		
Bird.ID	0.73	1.48	
***Correlation structure AR* (1)**	***Phi1***			
Formula: ~ Section number|Bird.ID	0.87			
***Model information***	***Observations***	***R*** ^**2**^ ***adj***.	***Scale est***.	
	369	0.401	2.19	

*Note*: As a reference for results interpretation, an effective degrees of freedom (edf) values of smooth terms represent the wiggles of the spline where a value of 1 indicates a straight line. The estimates, standard error and t‐value characterize the model's fixed effects. The treatment predictor indicates the average difference between the control and MeHg exposed groups. Final model's significant variables (*p* < 0.05) are displayed in bold. Results from the model including the treatment × week interaction are presented in Table S3.

### 3.4 Cloacal protuberance

**FIGURE 4:**
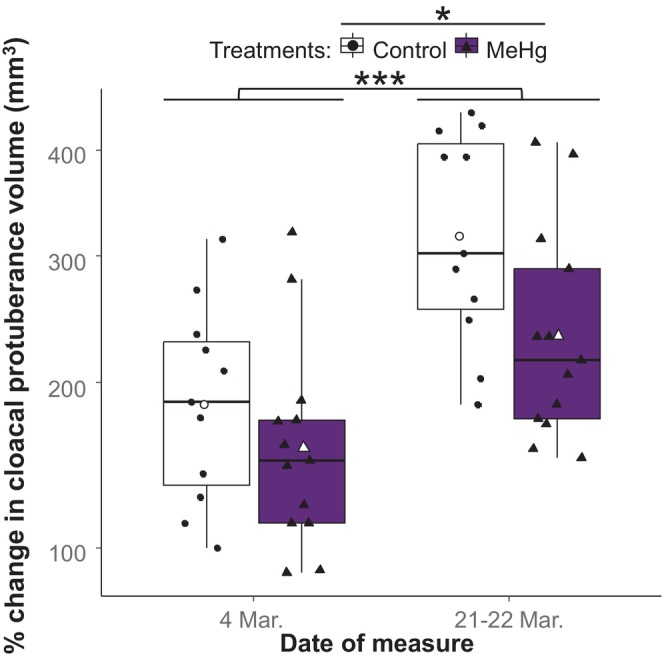
Change (%) in cloacal protuberance volume on square root scale, compared with pre‐photostimulation measures (18 February), in relation to date and MeHg treatment. Boxplots indicate 25th, 50th, and 75th percentiles and whiskers indicate range, with individual horizontally jittered data points overlaid. The boxplot color and symbols' shape indicate the individual's treatment, where the control group is indicated by black circles in white boxplots (*n* = 11), and the MeHg exposed group is indicated by black triangles in purple boxplots (*n* = 13). The white symbols indicate the mean volume change for each treatment group. The asterisks indicate significant effect of week of measure and MeHg treatment assessed in males only (*p < 0.05; ***p < 0.001). See Table 3 for the statistical model output.

Cloacal protuberance was not affected by the treatment × time interaction (Table S4), but a model without this interaction included the significant effect of treatment and of week of measure (Table 3). Cloacal protuberance volume increased with time, and MeHg‐exposed males had a smaller increase in volume than control males (Figure 4).

**TABLE 3:** Result of final lme model on males percentage of change in cloacal protuberance volume compared with pre‐photostimulation measure (18 February).

% Change in cloacal protuberance volume, simplified, first hypothesis					
*Predictors*	*Estimates*	*SE*	*95% CI*	*Statistic*	*p*
**(Intercept)**	**13.99**	**0.70**	**12.56 to 15.41**	**20.00**	**<0.001**
**Treatment [MeHg]**	**−1.80**	**0.84**	**−3.54 to −0.053**	**−2.14**	**0.044**
**Week [21–22 March]**	**3.45**	**0.65**	**2.11 to 4.80**	**5.31**	**<0.001**
***Random effects***	***Variance***	***SD***			
Bird.ID *τ*_00_	1.67	1.29			
Residuals *σ* ^2^	5.085	2.26			
ICC	0.25				
Observations/*N*	48/24				
Marginal *R* ^2^/Conditional *R* ^2^	0.36/0.52				

*Note*: The brackets [21–22 March] or [MeHg] indicates which level within a factor signal is compared with the reference group (e.g., compared with 4 March or to control birds). Final model's significant variables (*p* < 0.05) are displayed in bold. Results from the model including the treatment × week interaction are presented in Table S4.

### 3.5 Testes and body tissues

In males, testes mass was not affected by treatment (*t*‐test: *t* = −1.18, *df* = 21.019, *p* = 0.25; mean ± *SD* of control: 80.25 ± 35.18 mg, MeHg: 98.15 ± 40.66 mg). Syrinx mass was also not affected by treatment (*t*‐test: *t* = −0.80, *df* = 20.27, *p* = 0.43; mean ± *SD* of control: 18.21 ± 2.41 mg, MeHg: 18.95 ± 2.14 mg). Finally, brain mass was not affected by treatment (*t*‐test: *t* = 0.20, *df* = 19.28, *p* = 0.85; mean ± *SD* of control: 0.70 ± 0.053 g, MeHg: 0.69 ± 0.043 g). The liver mass model indicated no effect of treatment (lm: estimate = 0.11; standard error = 0.073; *t*‐value = 1.56; *p* = 0.13; control: 0.84 ± 0.18 g, MeHg: 0.96 ± 0.20 g), but a positive relationship with body mass before euthanasia (lm: estimate = 0.050; standard error = 0.020; *t*‐value = 2.48; *p* = 0.022). The model was significant (lm model: *F*_2,21_ = 4.66, residuals = 0.18, adj‐*R* ^2^ = 0.24; *p* = 0.022).

### 3.6 Brain GnRH

There was no significant effect of treatment on total GnRH‐ir cell numbers (*t* = 0.59, *df* = 14, *p* = 0.57) though a trend did occur suggesting lack of statistical power (Figure 2C). In a second analysis, we used a GAMM model to determine if GnRH‐ir cell variation along the rostral‐caudal axis differed with treatment. The number of GnRH‐ir cells was not affected by the interaction of brain section × treatment, which was significant, or the main effect of treatment, but did significantly vary with brain section number (Table 4). Control birds had a similar rostral peak in GnRH‐ir cells compared with MeHg exposed birds (Figure 2D).

**TABLE 4:** Results of GAMM model on GnRH‐ir cell count according to section number.

GnRH‐ir cell count per brain section				
*Parametric coefficients*	*Estimates*	*SE*	*t value*	*p*
**(Intercept)**	**2.17**	**0.11**	**19.45**	**<0.001**
Treatment	−0.1	0.16	−0.64	0.52
***Smooth parameters***		***edf***	***F value***	***p***
Section number		4.908	8.014	<0.001
Section number × Treatment [MeHg]		1.98	0.81	0.46
***Random effects***	***Intercept***	***Residual***		
Bird.ID	0.00016	0.95		
***Correlation Structure: ARMA*(*2***, ***0*)**	***Phi1***	***Phi2***		
Formula: ~ Section number|Bird.ID	0.44	0.17		
***Model information***	***Observations***	***R*** ^**2**^ ***adj***.	***Scale est***.	
	240	0.303	0.89	

*Note*: The brackets [MeHg] indicates which level within a factor is compared with the reference group (i.e., compared with control birds). Final model's significant variables (*p* < 0.05) are displayed in bold.

## 4 DISCUSSION

In this study, we tested if MeHg exposure during the overwinter phase could affect reproductive onset in song sparrows. Contrary to our predictions, the results indicated very little MeHg effect on the timing of spring activation of the HPG axis (GnRH, testes mass, testosterone) and body condition. Due to the THg measured in blood and the results of our team's prior studies where slightly lower MeHg doses affected molt, migratory behavior, and hormones in autumn,^49^, ^58^ we are confident that the exposure dose was high enough to elicit physiological responses. We were also able to detect seasonal physiological modifications associated with photostimulation and reproductive onset in all measures. Hence, this suggests that songbirds appear capable of buffering the effects of prior winter MeHg exposure so as to not delay reproductive onset in spring. It also suggests that documented MeHg negative effects on reproduction^35^, ^36^, ^37^, ^38^, ^39^, ^40^, ^41^, ^42^, ^43^, ^44^, ^45^, ^47^ would mainly be caused by MeHg exposure on breeding grounds, instead of carry‐over effects from exposure on wintering grounds. However, despite our generally null results, there is a potential for MeHg carry‐over effects through disruption of cloacal protuberance size.

Consistent with our results, several authors did not find MeHg effects on androgens such as testosterone.^79^, ^80^, ^81^, ^82^ However, others did,^83^, ^84^ suggesting that MeHg actions on this hormone may be age‐, species‐, or season‐dependent. Song sparrows could also be a species with very high resistance to deleterious MeHg effects on the HPG axis. Song sparrows generally breed near wetlands where mercury is methylated and enters foodwebs,^85^, ^86^, ^87^ so they may be well adapted to coping with moderate levels of MeHg. Alternatively, to resisting the effects of MeHg, our song sparrows may have repaired or compensated for any effects during the post‐exposure period (21 days on the last cloaca measurement). However, this seems unlikely since MeHg‐induced physiological and behavioral disruptions were observed up to 2 months post‐exposure in the same species.^49^

The general lack of MeHg effects in this study contrasts with our prior work using the same species, with diet manipulation and similar sample sizes, in which we observed a variety of carry‐over effects of MeHg exposure during the summer on subsequent molt, glucocorticoid activity, and migratory behavior.^49^, ^54^ In contrast to these multiple effects of summer MeHg exposure, our winter exposure had only minimal effects on breeding onset (cloaca size). Perhaps the fact that birds were fed ad libitum, or that we only measured the first 3 weeks of photostimulation (necessitated by the COVID‐19 pandemic shut down), limited our ability to detect carry‐over effects on reproductive onset. Indeed, the field stressful conditions could be associated with higher sensitivity to MeHg effects for the similar exposure doses compared with controlled experiments,^88^, ^89^, ^90^, ^91^ although unpredictable food stress does not appear to interact with MeHg effects in this species, except for glucocorticoid‐associated measures.^49^, ^54^ It also seems likely that, given the importance of reproductive timing to fitness, and as song sparrows have been exposed to MeHg throughout their evolution, this species may be able to buffer their reproductive competence against the deleterious effects of mercury. Indeed, previous work on zebra finches suggests strong selection for resistant genotypes under MeHg contamination.^92^, ^93^ Future work should test birds using different timelines and using both males and females to clarify the extent to which song sparrow reproductive neuroendocrine systems can cope with MeHg.

The significant effects of MeHg on cloacal protuberance size suggest that MeHg exposure in winter may induce an energetic cost that could ultimately lead to a reduction in mating success, even if the timing of reproductive onset was not affected. Indeed, a large cloacal protuberance is associated with increased sperm competition,^94^ but further work would be required to determine how cloaca size might relate to reproductive success in this species.

While MeHg reduces GnRH production and gene expression in other taxa (fish and mammals) and in chicken cells,^95^, ^96^, ^97^ such in vivo effects in birds were unknown. Interestingly, MeHg exposure had no overall effect on total GnRH‐ir cell numbers, or on the distribution of cells along the rostral–caudal axis of the hypothalamus (Figure 2D). It may be possible that there is more biological variability of GnRH neurons within a brain due to the coronal sections, than variability between the treatment groups. Thus, the variable “section number” could have captured the majority of the model variability or statistical power, especially with the corARMA function, that may not have left enough sensitivity to for the GAMM model to detect the treatment main effect. But, because the *t*‐test on all GnRH cells count per birds showed similar results, the GAMM model results are likely representative of the biological phenomenon occurring in the birds. This result may explain why the other components of the HPG axis were not affected in our study despite other studies finding effects of MeHg exposure on HPG axis components. This suggests high resistance of this neuroendocrine component to deleterious effects of MeHg.

## 5 CONCLUSION

In conclusion, we found no effect of MeHg exposure during the winter period on the reproductive onset of male birds. However, this study's duration of post‐exposure observations was short, and MeHg did directly disrupt cloacal protuberance, which could affect breeding success in the longer term. Future studies should incorporate more long‐term assessments of MeHg effects post‐exposure and assess sex differences in exposure sensitivity.

## AUTHOR CONTRIBUTIONS

**Claire L. J. Bottini:** Conceptualization; methodology; data curation; investigation; formal analysis; supervision; visualization; writing – original draft; writing – review and editing. **Calista J. Henry:** Data curation; formal analysis; investigation; writing – review and editing. **Scott A. MacDougall‐Shackleton:** Conceptualization; validation; resources; writing – review and editing; supervision; project administration; funding acquisition.

## FUNDING INFORMATION

This work was supported by the Natural Sciences and Engineering Research Council (NSERC) Discovery Grants to Scott A. MacDougall‐Shackleton (RGPIN‐2018‐05658) and a project support grant from the British Society for Neuroendocrinology acquired in 2019.

## CONFLICT OF INTEREST STATEMENT

The authors declare that they have no conflict of interest.

## PEER REVIEW

The peer review history for this article is available at https://www.webofscience.com/api/gateway/wos/peer-review/10.1111/jne.70027.

## ETHICS STATEMENT

All applicable international, national, and/or institutional guidelines for the care and use of animals were followed. All birds were captured under permission from the Canadian Wildlife Service and Environment and Climate Change Canada (Scientific Collection Permit CA 0244). All animal procedures were approved by The University of Western Ontario Animal Use Subcommittee (protocol no. 2017‐161).

## Supporting information


**Data S1.** Supporting Information.

## Data Availability

Datasets and R code presented in this article are available from the Mendeley data repository (Reserved DOI: 10.17632/4sdmfy98xc), or by contacting the corresponding author.
