# Supplementary material for: Effects of wintering under methylmercury exposure on spring reproductive onset in song sparrows (Melospiza melodia)
Source: J Neuroendocrinol. 2025 Apr 21;37(6):e70027. doi: 10.1111/jne.70027 (PMC12145943; doi:10.1111/jne.70027)
Supplement: Supplementary file 1 — Data S1. Supporting Information. [file JNE-37-e70027-s001.docx]

***Supplementary information for:***

Effects of wintering under methylmercury exposure on spring reproductive onset
in song sparrows (*Melospiza melodia*)

Claire L.J. Bottini^1,2*^, Calista J. Henry^2,3^ & Scott A. MacDougall-Shackleton^2,3^

Author affiliation:

^1^The University of Western Ontario, Department of Biology, 1151 Richmond St., London Ontario, N6A 5B7

^2^Advanced Facility for Avian Research, University of Western Ontario, London, Ontario, Canada

^3^The University of Western Ontario, Department of Psychology, 1151 Richmond St., London Ontario, N6A 5C2

^*^Corresponding author: cbottin@uwo.ca,

**This document contains:**

- Text S1 and Table S1: Synthetic food preparation

- Table S2: Plasma testosterone concentration results of interactive effect of MeHg and time.

- Table S3: Bird’s mass variation results of interactive effect of MeHg and time.

- Table S4: Males percentage of change in cloacal protuberance results of interactive effect of MeHg and time.

- Figure S1: Male bird’s mass (g) variation in relation to time and treatment

**Text S1: Synthetic food preparation:**

To prepare a 5 mg.kg^−1^methylmercury (MeHg) stock solution we mixed 0.645 mL of 1000 mg.kg^−1^ standard MeHg solution (Methylmercury Chloride, MeHgCl, Alfa Aesar #33553) into 129.355 mL of double distilled water. This solution was then mixed into 2700 g of uncontaminated agar-based food. During the 7 months of experiment, each food batches (methylmercury contaminated and uncontaminated) were made once or twice a week. We measured THg of each batch made during the exposure period as described in the main methods.

To prepare agar-based food, we first oven dried 300g of corn starch and 52 g of agar at 60°C for >1 hour. To make the diet, we mixed 1699 mL of boiling tap water with 45 g of food grade agar (G-Biosciences, #RC-004). The agar solution was hand mixed at room temperature for 30 sec before being heated up to boiling again for about 70-90 sec and then added it to a batch containing a mixture of 7 components (Table S1). Boiled agar solution and other components were thoroughly blended for 1 min. We then transferred the blended mixture into a plastic container and allowed to cool down for 5 min at room temperature before adding either 130 mL of 5 mg.kg^−1^ MeHg stock solution or 130 mL of distilled water to make the contaminated and uncontaminated food respectively. With a plastic spatula, we carefully mixed the agar food and water solution. Once fully homogenized, we let it cool down in a fume hood for about 1h before we closed the labelled container and stored it in a refrigerator at 4 °C.

**TABLE S1**. Composition of synthetic agar-based diet, uncontaminated and MeHg contaminated.

| Components | 0 mg.kg^−1^  (Uncontaminated) | 0.22 mg.kg^−1^MeHg  (Contaminated) | % dry mass | % wet mass |
| --- | --- | --- | --- | --- |
| Corn starch (g) | 261 | 261 | 30 | 9.7 |
| Dextrose (g)^a^ | 261 | 261 | 30 | 9.7 |
| Casein (g)^b^ | 117 | 117 | 13.4 | 4.3 |
| Canola oil (g) | 92 | 92 | 10.6 | 3.4 |
| Salt mixture (g)^d^ | 51 | 51 | 5.8 | 1.9 |
| Agar (g)^f^ | 45 | 45 | 5.2 | 1.7 |
| Celufil (g)^e^ | 28 | 28 | 3.2 | 1.0 |
| Vitamins (g)^c^ | 16 | 16 | 1.8 | 0.6 |
| Water (mL) | 1699 | 1699 | - | 62.9% |
| Additional water (mL) | 130 | 129.355 | - | 4.8% |
| 1000 mg.kg^−1^ MeHg solution (mL) | - | 0.645 | - |  |

Product information: a) Dextrose or D-Glucose Anhydrous (Granular Powder/Certified ACS, Fisher Scientific #D16-10); b) Casein, high nitrogen, Ultrapure (Alfa Aesar by Thermo Fisher Scientific, #J12845-Q1); c) AIN-76 Vitamin mixture (MP Biomedicals, #ICN90545401); d) Briggs N Salt Mixture (MP Biomedicals, #902834); e) Cellulose fibrous (Sigma-Aldrich, #C6288); f) Agar (G-Biosciences, #RC-004).

TABLE S2: Result of glmm model on plasma testosterone concentration, corresponding to our second hypothesis (interactive effect of MeHg and time). Indication of [MeHg] and [month] signals which factor group of the data is compared to the reference group (e.g., control treatment or month of January). Final model’s significant variables (p<0.05) are displayed in bold.

| Plasma testosterone levels variation, model for second hypothesis | | | | | |
| --- | --- | --- | --- | --- | --- |
| *Predictors* | *Estimates* | *SE* | *95% CI* | *Statistic* | *P* |
| **(Intercept)** | **0.22** | **0.008** | **0.21_0.24** | **26.58** | **< 0.001** |
| Treatment [MeHg] | -0.013 | 0.011 | -0.035_0.0090 | -1.15 | 0.25 |
| Month [February] | 0.006 | 0.011 | -0.015_0.027 | -0.57 | 0.57 |
| **Month [March]** | **-0.051** | **0.0094** | **-0.070_-0.034** | **-5.51** | **< 0.001** |
| Treatment [MeHg] × Month [February] | 0.0086 | 0.014 | -0.020_0.037 | 0.60 | 0.54 |
| Treatment [MeHg] × Month [March] | 0.0048 | 0.012 | -0.020_0.029 | 0.38 | 0.70 |
| *Random Effects* | *Variance* | *SD* |  |  |  |
| Bird.ID τ_00_ | <0.001 | 0.0086 |  |  |  |
| Residuals σ^2^ | 0.014 | 0.12 |  |  |  |
| Intraclass-correlation coef. (ICC) | 0.27 | | | | |
| Observations / N | 72 / 24 | | | | |
| Marginal R^2^ / Conditional R^2^ | 0.54 / 0.59 | | | | |

TABLE S3: Result of GAMM model on bird’s mass variation, corresponding to our second hypothesis (interactive effect of MeHg and time). The effective degrees of freedom (edf) values of smooth terms represent the wiggles of the spline where a value of 1 indicates a straight line. The estimates, standard error and t-value characterize the model’s fixed effects. The treatment predictor indicates the average difference between the control and MeHg exposed groups. Final model’s significant variables (p<0.05) are displayed in bold.

| Bird’s mass variation | | | | |
| --- | --- | --- | --- | --- |
| *Parametric coefficients* | *Estimates* | *Std. Error* | *t value* | *P* |
| **(Intercept)** | **23.28** | **0.23** | **101.35** | **<0.001** |
| Treatment | 0.10 | 0.32 | 0.32 | 0.75 |
| *Smooth parameters* |  | *edf* | *F value* | *P* |
| **Week** |  | **10.16** | **29.152** | **<0.001** |
| Week × Treatment |  | 1.00 | 0.198 | 0.66 |
| *Random Effects* | *Intercept* | *Residual* |  |  |
| Bird.ID | 0.70 | 1.50 |  |  |
| *Correlation structure AR(1)* | *Phi1* |  |  |  |
| Formula: ~ Section number \| Bird.ID | 0.87 |  |  |  |
| *Model information* | *Observations* | *R^2^ adj.* | *Scale est.* |  |
|  | 369 | 0.4 | 2.25 |  |


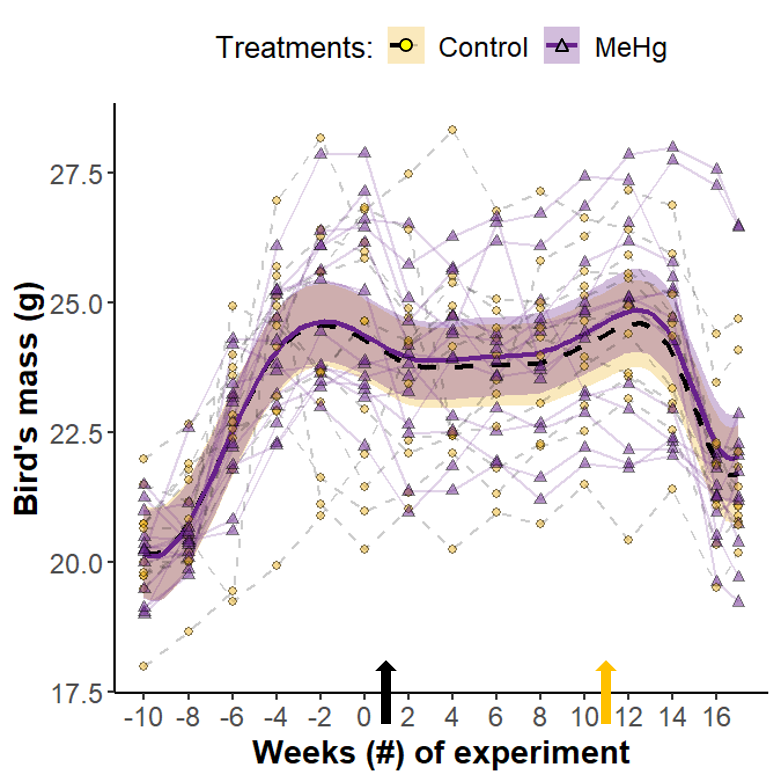


FIGURE S1: Male bird’s mass (g) variation in relation to time and treatment. Nonparametric splines were fitted with the predictions of the GAMM models at 10 knots and 95 % confidence interval. Lines type and colors as well as symbols shape and colors indicate the treatment groups, where the control group is represented by dashed line and white circles (n = 12), and the MeHg exposed group is represented by solid line and purple triangles (n = 13). The black vertical arrow indicates the start of MeHg exposure on 2 December, while the yellow vertical arrow indicates the end of exposure and time of photostimulation on 2 March. The first time point of measure (week # -10) corresponds to the bird’s mass upon its arrival into captivity between the 5 to 23 September 2019, then, starting on 2 October (week # -8) birds were weighted once every two weeks until the end of the experiment on 27-29 March (week # 17). Connected symbols represent individual bird ID.

TABLE S4: Result of final lme model on males percentage of change in cloacal protuberance volume compared to pre-photostimulation measure (18 February). The brackets [21-22 March] or [MeHg] indicates which level within a factor signals is compared to the reference group (e.g., compared to 4 March or to control birds). Final model’s significant variables (p<0.05) are displayed in bold.

| % change in cloacal protuberance volume, model for second hypothesis | | | | | |
| --- | --- | --- | --- | --- | --- |
| *Predictors* | *Estimates* | *Std. Error* | *95% CI* | *Statistic* | *P* |
| **(Intercept)** | **13.61** | **0.78** | **12.026 – 15.19** | **17.38** | **<0.001** |
| Treatment [MeHg] | -1.099 | 1.064 | -3.25 – 1.048 | -1.033 | 0.307 |
| **Week [21-22 March]** | **4.21** | **0.96** | **2.22– 6.20** | **4.39** | **<0.001** |
| Treatment [MeHg] × Week [21-22 March] | -1.40 | 1.30 | -4.096 – 1.31 | -1.072 | 0.30 |
| *Random Effects* | *Variance* | *Std. Dev.* |  |  |  |
| Bird.ID τ_00_ | 1.69 | 1.30 |  |  |  |
| Residuals σ^2^ | 5.052 | 2.25 |  |  |  |
| ICC | 0.25 |  | | | |
| Observations / N | 48 / 24 |  | | | |
| Marginal R^2^ / Conditional R^2^ | 0.37 / 0.53 | | | | |
